# Chloroquine and beyond: exploring anti-rheumatic drugs to reduce immune hyperactivation in HIV/AIDS

**DOI:** 10.1186/s12977-015-0178-0

**Published:** 2015-06-18

**Authors:** Andrea Savarino, Iart Luca Shytaj

**Affiliations:** Department of Infectious, Parasitic and Immune-Mediated Diseases, Istituto Superiore di Sanità, Viale Regina Elena, 299, 00161 Rome, Italy

**Keywords:** Immune activation, HIV latency, Chloroquine, Hydroxychloroquine, Anti-rheumatic, Clinical trials, Auranofin

## Abstract

The restoration of the immune system prompted by antiretroviral therapy (ART) has allowed drastically reducing the mortality and morbidity of HIV infection. However, one main source of clinical concern is the persistence of immune hyperactivation in individuals under ART. Chronically enhanced levels of T-cell activation are associated with several deleterious effects which lead to faster disease progression and slower CD4^+^ T-cell recovery during ART. In this article, we discuss the rationale, and review the results, of the use of antimalarial quinolines, such as chloroquine and its derivative hydroxychloroquine, to counteract immune activation in HIV infection. Despite the promising results of several pilot trials, the most recent clinical data indicate that antimalarial quinolines are unlikely to exert a marked beneficial effect on immune activation. Alternative approaches will likely be required to reproducibly decrease immune activation in the setting of HIV infection. If the quinoline-based strategies should nevertheless be pursued in future studies, particular care must be devoted to the dosage selection, in order to maximize the chances to obtain effective in vivo drug concentrations.

## Background

The quest for clinical candidates to counteract immune activation has become a “hot topic” in AIDS research, because HIV infection is characterized by malignant immune hyperactivation which correlates with disease progression and poor response to antiretroviral therapy (ART) [1–5]. Moreover, immune hyperactivation is also regarded as a major obstacle to a cure for AIDS [6].

In the beginning of the millennium, an article authored by one of us launched chloroquine as a tool to inhibit viral replication and the related malignant immune activation associated with some viral diseases [7]. This article sparked a new wave of studies, in that it extended a theory, previously designed for HIV/AIDS [8], to other viral diseases characterized by excessive immune activation. As will be discussed below, by accumulating in the acidic organelles, chloroquine exerts both direct antiviral effects on enveloped viruses and decreases activation of several cell types involved in the immune response. Chloroquine has since shown promise in preclinical studies (both in vitro and in vivo), as a therapeutic agent against emerging viruses such as MERS CoV [9]. Of note, chloroquine has been indicated as a promising candidate for filovirus treatment [10], especially during the latest Ebola epidemic [11, 12]. In two studies out of three, chloroquine showed antiviral activity in mice at the maximum tolerated dose [10, 13, 14], thus rendering this drug an interesting agent for further testing of combination anti-Ebola therapies. However, the effects of chloroquine and its hydroxyl analogue hydroxychloroquine, on HIV infection, i.e. the initial target for the repurposing of these drugs, have remained controversial. On the one hand, based on the results of some earlier clinical trials, chloroquine/hydroxychloroquine has been recently re-suggested as a promising candidate to restrict the HIV-related immune activation [15, 16]. On the other hand, the results from the latest clinical trials indicate that chloroquine/hydroxychloroquine has no beneficial effect on immune activation [17, 18].

We here provide a state of the art of the studies investigating the use of chloroquine/hydroxychloroquine as a therapeutic tool for HIV/AIDS and suggest the possible biological grounds for the clinical results obtained. Moreover, we describe the reasons why our group decided to proceed further with strategies based on another drug, i.e. auranofin, which shares with chloroquine an anti-rheumatic effect [19].

## Immune activation in HIV/AIDS

Several reviews have recently been published on immune activation in HIV infection [6, 16, 20, 21]. Briefly, immune hyperactivation, commonly measured as the expression levels on peripheral blood lymphocytes of markers such as HLA-DR, CD38, or CD69 correlates with, and also predicts, disease progression (reviewed in [22, 23]). Immune activation gradually decreases following therapy initiation [24] and is maintained high in immunological non-responders, who are individuals maintaining low CD4 counts despite prolonged exposure to ART [3, 4]. While the initial studies were focused on the relationship between disease progression and activation of CD8^+^ T-cells [1], later studies better concluded that there is a broader relationship between disease progression and immune hyperactivation, involving also CD4^+^ T-cells [5, 25] and innate immunity [26].

Immune activation and viral replication are believed to be mutually enhanced in a vicious circle. The virus, recognized by the immune system as non-self, induces immune activation, which, in turn, fuels viral replication by furnishing to the virus material to synthesize the different viral components. For example, lymphocyte activation increases the cytoplasmic levels of deoxyribonucleotides necessary for viral DNA synthesis by reverse transcriptase [27]. This vicious circle may still persist in anatomical compartments incompletely penetrated by ART.

**Figure 1:**
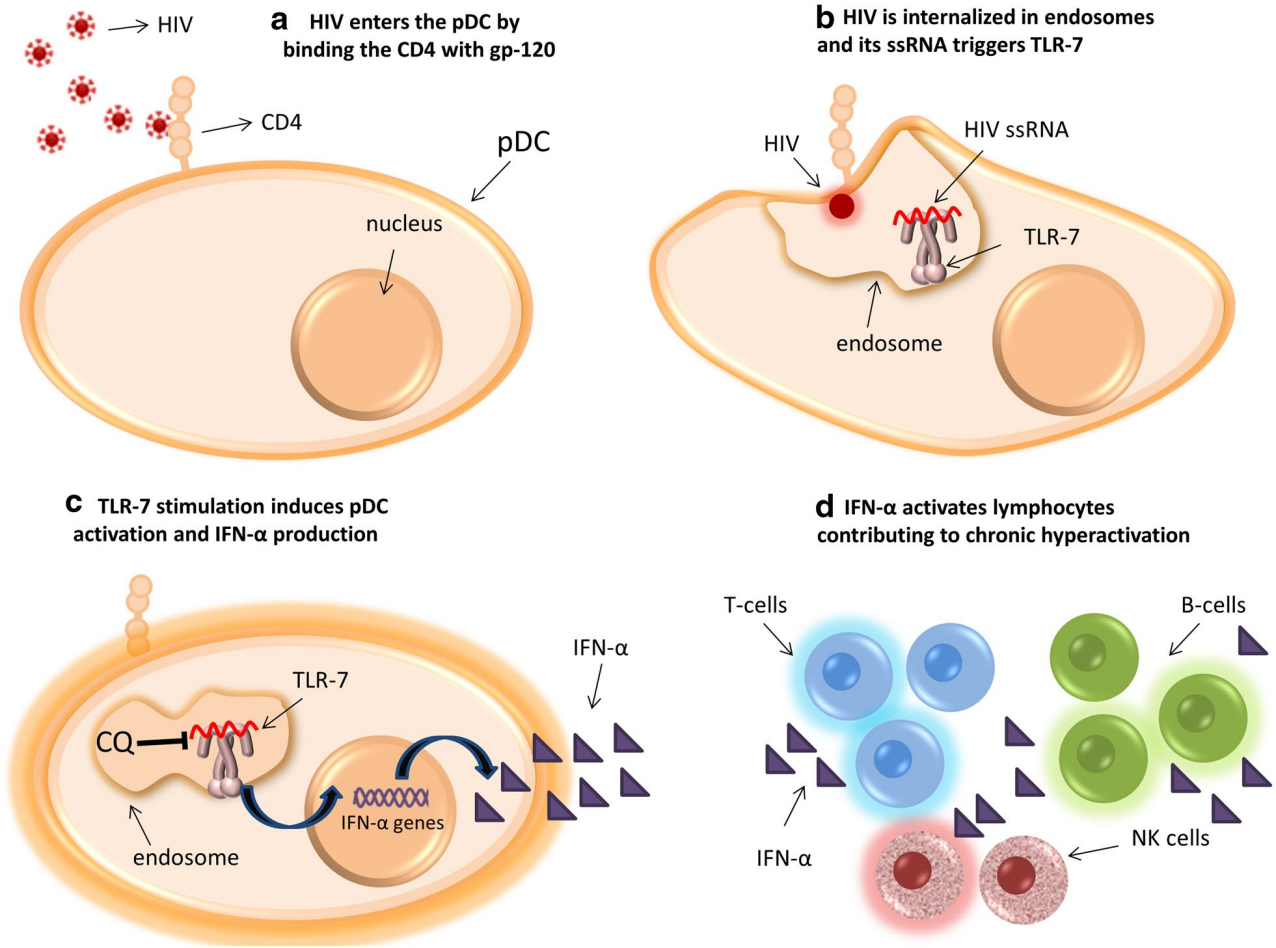
Mechanistic model of HIV-induced persistent immune-activation. **a** HIV enters CD4-expressing plasmacytoid dendritic cells (pDCs); **b** the virus is endocytosed, decapsided and its RNA is recognized by toll-like receptor 7 (TLR-7); **c** stimulation of TLR-7 prompts a signaling cascade inducing IFN-α transcription in the nucleus; **d** production of IFN-α favors activation of several cell subsets such as T, B and natural killer (NK) lymphocytes. Chloroquine (CQ) is postulated to reduce the efficiency of this mechanism by accumulating in endosomes and decreasing HIV-mediated TLR-7 signaling [44].

HIV-induced immune activation is not limited to specific immunity, but exerts its effects on innate immunity as well. HIV-1 was shown to activate plasmacytoid dendritic cells (pDCs), which, differently from myeloid dendritic cells (the most potent antigen-presenting cells in the body), induce innate antimicrobial immunity by producing type I interferons (Figure 1) [26]. pDCs internalize HIV-1 through viral envelope/CD4 interactions, and the internalized virus activates these cells mainly through toll-like receptor 7 (TLR-7) signaling (Figure 1). Comparative pathology corroborates the hypothesis that over-stimulation of this pathway may be associated with deleterious effects. Sooty mangabeys (*Cercocebus atys*), which can be infected by a simian homolog of HIV (i.e. simian immunodeficiency virus, SIV) but do not develop AIDS, display weak IFN-α production upon stimulation with TLR-7 antagonists [28]. On the contrary, rhesus macaques (*Macaca mulatta*), which do progress to AIDS, produce high amounts of IFN-α when their pDCs are subjected to the same stimuli [28]. Moreover, another species displaying nonpathogenic SIV infection, i.e. the African green monkey (*Chlorocebus aethiops*), is characterized by an efficient control of IFN-α production following acute infection [29].

pDCs decrease in peripheral blood during progression to AIDS, because, upon activation, they migrate to the lymphoid tissue [30]. As a huge number of cells reside in the gut-associated lymphoid tissue (GALT), according to the microbial translocation theory, the intestinal mucosa damaged by the consequent inflammation may become permeable to products of the gut microbiome which further enhance HIV-related immune hyperactivation [31, 32].

Finally, immune activation is one primary driver of both generation and maintenance of the viral reservoir, which is mainly constituted by latently infected, central and transitional memory CD4^+^ T-cells (henceforth T_CM_ and T_TM_, respectively) [33]. Also in this case, comparative pathology has provided clues for understanding this phenomenon. It was shown that CD4^+^ T_CM_ cells from sooty mangabeys express, upon activation, low levels of CCR5, the main coreceptor for virus entry into cells, thus limiting infection of this important cellular compartment [34]. Instead, activated T_CM_ cells from AIDS-developing species, such as humans and rhesus macaques, up-regulate the levels of CCR5 to a higher extent than cells from sooty mangabeys, thus facilitating the generation of a consistent viral reservoir [34]. After these cells become quiescent, viral replication switches off, and latently infected, HIV-reservoir cells proliferate through low-level antigenic stimulation (T_CM_) or IL-7-driven homeostatic proliferation (T_TM_) [33]. Both processes are enhanced by generalized immune activation.

## Mechanisms of action of chloroquine

**Figure 2:**
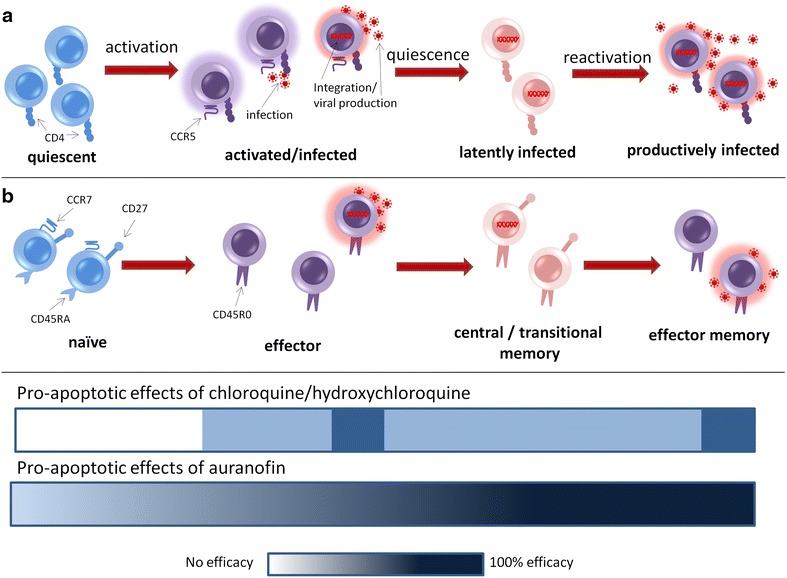
Comparison of the susceptibility to chloroquine/hydroxychloroquine and auranofin of the cellular subsets involved in HIV production and persistence. Shown in the figure is a schematic depiction of **a** activation and **b** differentiation stages of CD4^+^ T-lymphocytes and their correlation with viral production, latency and viral reactivation. Both chloroquine/hydroxychloroquine and auranofin can influence these transitions by exerting a pro-apoptotic effect, the efficacy of which is graphically exemplified by the intensity of the *blue color* in the corresponding *rectangles*. Efficacy gradients are based on data derived from Refs. [45, 48, 50].

Multiple in vitro effects of chloroquine could support its possible use as a modulator of immune activation in HIV/AIDS:

1. Chloroquine and its hydroxyl analogue hydroxychloroquine were shown in several studies to inhibit HIV-1 replication (reviewed in: [7]). The effects of these quinolines, mainly due to the induction of a defect in the maturation of the viral envelope glycoprotein gp120 [35, 36], might mimic the effects of broadly neutralizing antibodies directed against the viral envelope, although the effects of these antibodies are weaker than those directed against the CD4-binding site [37]. These effects are additive to those of non-nucleosidic reverse transcriptase inhibitors (NNRTIs) and synergistic to those of protease inhibitors (PIs) [38]. As quinoline drugs accumulate in lymphoid tissues [39], they might decrease ongoing viral replication during ART in anatomical sanctuaries and, consequently switch off one of the main drivers of immune activation. Chloroquine is also an inhibitor of P-glycoprotein (P-gp) and multidrug resistance proteins (MRPs) [40, 41], cell surface glycoproteins which extrude several antiretroviral drugs to the extracellular medium. In line with this evidence, chloroquine was shown to increase the intracellular levels of PIs [38]. The effects of chloroquine in combination with NRTIs are instead controversial: some reported an additive effect [42], while others did not detect it [43]. The combined effects of chloroquine and integrase inhibitors are as yet unknown.
2. Chloroquine accumulates in phagosomes of pDCs and inhibits their HIV-induced activation [44]. It might therefore impact on innate immunity-induced immune hyperactivation.
3. A recent study showed that hydroxychloroquine selectively induces apoptosis in the memory T-cell compartment (CD45RA^−^ CD45RO^+^) [45]. As, upon activation, naïve T-cells (CD45RA^+^ CD45RO^−^) acquire a CD45RA^−^ CD45RO^+^ phenotype, the “antimemory” effect should limit immune activation (Figure 2) [46]. There is growing consensus that induction of apoptosis in the memory T-cell compartment might have a detrimental effect on the viral reservoir [47–49]. In this light, chloroquine/hydroxychloroquine should have an anti-reservoir potential. This view is supported by another recent study which shows that chloroquine sensitizes to apoptosis the latently infected cells upon viral reactivation, likely by removing the anti-apoptotic effect of the virus structural *gag* gene products [50]. These effects are potentially interesting, since it has been well demonstrated that viral reactivation from latency does not necessarily result in cell death [51].

## In vivo effects of chloroquine/hydroxychloroquine: preclinical models

The macaque AIDS model is an important tool for preclinical assessment of strategies aimed at treating HIV/AIDS [52]. To our knowledge, chloroquine has been tested in this model on two occasions.

In a first study, chloroquine (25 mg every other day for 30 days, i.e. a cumulative dosage comparable to that administered to humans with rheumatioid arthritis) was administered to three Chinese rhesus macaques infected with the simian HIV-homologue, SIVmac_251_ [53]. Although a decrease in activated pDCs was shown, no effects were seen on viral load and CD4^+^ and CD8^+^ T-cell activation (measured as CD38 expression) [53].

As the immune activation set point is established during acute infection [4], Vaccari et al. [54] treated with chloroquine (18.7 mg/day for 112 consecutive days) seven SIVmac_251_-infected rhesus macaques during the viral load peak that characterizes acute infection. Apart from an unexpected, although transient, increase in the expression of interferon-regulated genes (perhaps not population-relevant as possibly driven by only one animal), no significant differences were reported in viral load and T-cell activation and proliferation (measured as expression of CD69 and Ki67, respectively) [54]. A trend was however noticed for maintenance of decreased levels of Ki67, CD69 and CCR5 in the gut of the chloroquine-treated animals, although the differences with values from the control group did not reach statistical significance. The effect of chloroquine in this simian model in the presence of ART is still unknown.

## In vivo effects of chloroquine/hydroxychloroquine: clinical trials

**Figure 3:**
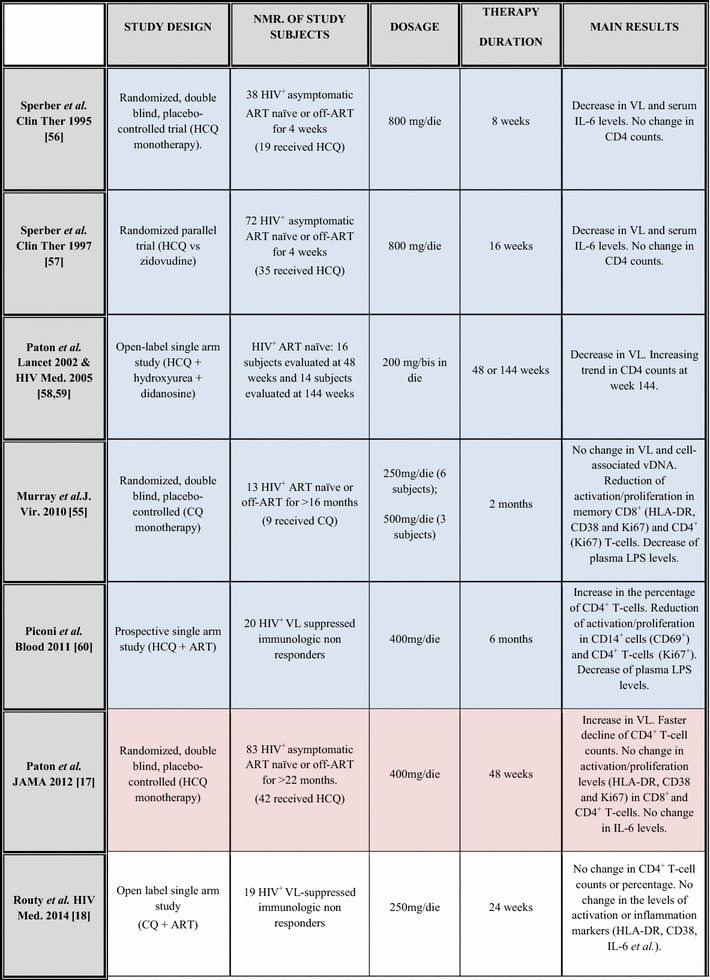
Published clinical studies evaluating the effects of chloroquine/hydroxychloroquine administration, alone or in combination with other drugs, in HIV infected subjects. Highlighted in *blue*, *red* or *white* are the studies that have reported a positive, negative, or neutral outcome of the therapy respectively. *CQ* chloroquine, *HCQ* hydroxychloroquine.

Chloroquine and hydroxychloroquine have so far been tested in several HIV clinical trials. The results summarized in Figure 3 support the hypothesis that the chloroquine/hydroxychloroquine dosage may be an important driver of at least partial clinical success.

Suppressive effects on immune activation by chloroquine were shown in the trial conducted by Murray et al. [55]. However, in this trial, the dosage administered was not the same for all individuals, some of them receiving 500 mg/die instead of 250 mg/die. It is thus possible that the statistical significance of the effects reported in this study was driven by the higher dosage of the drug. This view is supported by a later study which tested chloroquine at 250 mg/die and failed to show any effect of the drug [18].

In two clinical trials conducted in the 1990s, Sperber et al. reported suppressive effects on immune activation (measured at that time as IL-6 production) and viral load in individuals treated with 800 mg of hydroxychloroquine/day (bioequivalent to 500 mg/day of chloroquine) [56, 57]. The other clinical trials testing hydroxychloroquine at a lower dosage (i.e. 400 mg/day) led to conflicting results. Earlier studies [58, 59] and the more recent study of Piconi et al. [60] reported significant effects on viral load [58], CD4 counts [59], and immune activation. [60]. Instead, a more recent clinical trial, randomized and double blind, showed disappointing results, even hinting at possibly deleterious effects of hydroxychloroquine on viral load and CD4 counts [17]. This trial was conducted in the absence of ART, and this might explain differences between this study and the study of Piconi et al., which was conducted on individuals under ART [60]. Another trial in ART-treated patients is currently ongoing and will provide more information on the effects of hydroxychloroquine (ClinicalTrials.gov identifier: NCT01232660).

The hydroxychloroquine levels show high inter-subject variability and, although individuals receiving the higher hydroxychloroquine dosages (800 and 1,200 mg/day) also showed significantly higher blood levels of the drug than those receiving 400 mg/die, the range of the blood concentrations was in part overlapping in the different dosage groups [61]. Chloroquine has similar pharmacokinetics [62]; therefore, not only the dosage but also individual differences in drug metabolism and distribution may explain the different conclusions of the aforementioned studies. A large clinical trial has recently been completed (ClinicalTrials.gov Identifier: NCT00819390) and its results can help to better represent the response of a population, thus abolishing the bias due to limited sample size. In this trial, however, chloroquine has been tested at 250 mg/day in the absence of ART; thus, in light of the results of the aforementioned clinical trials and considerations derived from basic science (see next paragraph), it is not surprising that the preliminary results released so far for this trial (https://clinicaltrials.gov/ct2/show/NCT00819390) do not show any significant effect of chloroquine on immune activation, viral load and CD4 counts.

## Lessons learnt from chloroquine/hydroxychloroquine use in HIV infection

Chloroquine/hydroxychloroquine-treated individuals display blood concentrations that are highly variable and only rarely exceed 10 or 20 µM, respectively [61, 62]. Therefore, at the steady state levels, these blood concentrations only in part overlap those at which a therapeutic effect is expected. For example, the EC_50_ of chloroquine on PBMC proliferation upon activation is, in general, ≥10 µM [63], and this value can explain the varying results obtained in the different clinical trials, with clearer effects associated with the higher drug dosages. Similarly, the pro-apoptotic effect of hydroxychloroquine on the memory T-cells is only moderate at the concentrations reachable in blood, especially in the lower range [45, 61]. The pro-apoptotic effect of chloroquine described by Li et al. on latently infected cells upon viral reactivation is instead more marked, although still partial, at the upper range of clinically achievable blood concentrations (5–10 µM) [50]. This effect could therefore be visible in vivo in terms of viral reservoir reduction, but only treating with high chloroquine dosages in the presence of suppressive ART. Moreover, to maximize the chances to obtain viral reservoir reduction in vivo, chloroquine treatment should be prolonged, as the events of virus reactivation from latency are rather rare (estimated as one event of transition from latency to productive infection every 10 mL of blood each day) [64].

The effect of chloroquine on pDC activation (see Figure 1) was initially observed in vitro by pre-incubating pDCs with 100 µM of chloroquine for 1 h [44]. This treatment results in intracellular concentrations comparable to those observed during chronic in vivo administration [65]. In this case, the in vitro effect is in line with the results of two in vivo studies [53, 60]. The use of chloroquine-related compounds with increased potency is yielding promising results in vitro [66], and it will be interesting to test the best-performing candidates in the simian AIDS model.

The effects of chloroquine/hydroxychloroquine on viral replication have been repeatedly shown in vitro at lower drug levels than those inducing the cellular effects [35, 36, 63, 65]. The blood concentration/EC_50_ ratio is however much narrower than those shown by antiretroviral drugs [63]. The antiretroviral effects of chloroquine/hydroxychloroquine may though become visible in anatomical sanctuaries of those individuals treated with PI-containing antiretroviral regimens. In any case, we recommend that chloroquine/hydroxychloroquine be tested at the highest recommended dosages in future HIV clinical trials.

Alternative/complementary interpretations of the results so far obtained are possible. For example, the effectiveness of the ART regimen employed may play a role in determining the magnitude of the effects (if any) observed following chloroquine/hydroxichloroquine addition. The study of Piconi et al. [60], showing some benefit in immunological non responders, may indicate that the effects of chloroquine may be visible only in some subsets of individuals with peculiar immunological characteristics, and that these effects can be hindered when immunologically non homogeneous cohorts are studied. In this regard, larger studies, with cohorts stratified according to immunological responsiveness to ART, could provide further information on the effects of chloroquine/hydroxychloroquine.

Another open question remains the influence of the duration of drug exposure, as it has been shown that chloroquine/hydroxychloroquine has cumulative effects [67]. As a proportion of HIV-infected patients in Africa may already be on chloroquine medication to prevent malaria, it might be worth examining the long-term effects of this treatment. In this regard, an ongoing phase III clinical trial will assess the long-term effects of chloroquine and trimethoprim-sulfamethoxazole phrophylaxis on survival and disease control in HIV-infected individuals with suppressed viral load and good clinical response to ART [68].

## Current and future directions: another approach based on antirheumatic therapy

Given the aforementioned problems in the pharmacokinetics of chloroquine/hydroxychloroquine, our group chose to follow a different, yet partly similar, approach to corroborate treatment of HIV/AIDS. Based on the feedback received from basic science studies and clinical trials that have been published throughout the years, we decided to use drugs the desired effects of which be striking in vitro at concentrations lower than the trough plasma concentrations in vivo. We also decided to re-direct our research on the basis of the plasma concentrations rather than on whole-blood concentrations (widely used for chloroquine/hydroxychloroquine), because we thought that the former might better mimic the tissue culture concentrations. The drug that we selected is the gold-based compound auranofin, the pharmacodynamics and pharmacokinetics of which are well known, due to its decade-long employment for treatment of rheumatoid arthritis [69].

The main rationale for the use of auranofin in our studies was its ability to target the central/transitional memory CD4^+^ T-cell compartment (Figure 2) [48, 70], which is known to harbor the main viral reservoir in patients receiving ART [33]. Auranofin is drastically active at sub-micromolar (i.e. ≤250 nM) concentrations, which are below those readily achievable in human plasma [71]. The administration of auranofin ultimately led to a reduction of the viral reservoir in ART-treated SIVmac251-infected macaques [70]. A review on our preclinical studies has recently been published [46] and the reader is addressed to it for further detail. Not surprisingly for a drug effective against an autoimmune disease such as rheumatoid arthritis, auranofin may as well be beneficial in terms of reduction of cell activation. In particular, the downregulation of the CD28 molecule induced by auranofin can disrupt the co-stimulatory signal often crucial for lymphocyte activation [48]. Moreover, apart from memory CD4^+^ T-cells, auranofin also targets the memory CD8^+^ T-cell compartment [48], i.e. a cellular subset known to be hyperactivated during HIV infection [2]. Interestingly, as described for hydroxychloroquine [60], auranofin was shown to disrupt in various cell lines the TLR-4 signaling [72], which is activated by bacterial lipopolysaccharides and likely constitutes another source of immune hyperactivation. In vitro data indicate that the impact of auranofin on lymphocyte activation may be mediated, at least in part, by modulation of oxidative stress [48]. Of note, the addition of a potent pro-oxidant drug, such as buthionine sulfoximine (BSO), increases the potency of auranofin, decreasing phytohemagglutinin-induced activation and expression of the α-chain of the IL-2 receptor [73]. This is in line with our preliminary data in SIVmac251-infected macaques, in which a combined regimen of ART, auranofin and BSO induced a functional cure-like condition following suspension of all therapies [74]. These observations provide proof of concept that drastically decreasing immune hyperactivation arrests SIV disease progression and turns the virus/immune system balance in favor of the latter. Clinical trials will be required to assess the potential of auranofin to decrease immune activation in ART-treated subjects.

Finally, other drugs used or proposed for treatment of rheumatoid arthritis might find a place in the treatment of HIV/AIDS. For example, the janus kinase inhibitors tofacitinib and ruxolitinib have shown a promising in vitro activity against HIV replication [75]. The ongoing in vivo studies on these compounds could provide an opportunity to analyze the effects of this treatment on viral replication and immune activation.

## Abbreviations

ART: antiretroviral therapy
AIDS: acquired immunodeficiency syndrome
BSO: buthionine sulfoximine
CCR5: C–C chemokine receptor type 5
CD: cluster of designation/differentiation
GALT: gut associated lymphoid tissue
HIV: human immunodeficiency virus
IL: interleukin
MERS CoV: middle east respiratory syndrome coronavirus
MRP: multidrug resistance protein
NRTI: nucleosidic reverse transcriptase inhibitor
NNRTI: non-nucleosidic reverse transcriptase inhibitor
pDC: plasmacytoid dendritic cell
P-gp: P-glycoprotein
PI: protease inhibitor
SIV: simian immunodeficiency virus
TLR: toll-like receptor
T_CM_: central memory T-cell
T_TM_: transitional memory T-cell
